# New image, same tactics: global tobacco and vaping industry strategies to promote youth vaping

**DOI:** 10.1093/heapro/daae126

**Published:** 2024-11-04

**Authors:** Christina Watts, Shiho Rose, Bronwyn McGill, Amelia Yazidjoglou

**Affiliations:** The Daffodil Centre, The University of Sydney, A Joint Venture with Cancer Council NSW, 1 King Street, Newtown, NSW, 2042, Australia; The Daffodil Centre, The University of Sydney, A Joint Venture with Cancer Council NSW, 1 King Street, Newtown, NSW, 2042, Australia; Prevention Research Collaboration, Sydney School of Public Health, Faculty of Medicine and Health, The University of Sydney, John Hopkins Drive, Camperdown, NSW, 2006, Australia; Charles Perkins Centre, The University of Sydney, John Hopkins Drive, NSW, 2006, Australia; Centre of Epidemiology for Policy and Practice, National Centre for Epidemiology and Population Health, Australian National University, 62 Mills Road, Acton, Australian Capital Territory, 2601, Australia

**Keywords:** commercial determinants of health, e-cigarettes, tobacco industry interference, public health, health policy

## Abstract

E-cigarette use (or vaping) is widespread in young people and is a rapidly growing public health problem. While the tobacco and vaping industry has promoted vaping as a smoking cessation aid for adults, the industry has strategically targeted young people through marketing and appealing designs to orientate a new generation of consumers to use their products. These strategies are not new and replicate what we have previously seen employed by the tobacco industry in past decades to maintain and grow their tobacco profits. We review the evidence on tobacco and vaping industry interference, highlighting the calculated and strategic use of interference tactics as a discourse to curb tobacco control efforts. We demonstrate how these tried and tested strategies are now being purposefully re-used in the context of vaping. As Australia is currently undergoing significant policy reforms for the access and retail of vaping products, we also provide a case study of the industry response played out in this contemporary landscape. Government and public health advocates are in a key position to be one step ahead in proactively tackling the vaping crisis. We recommend that continued monitoring of industry activities and strategies, achieving political transparency and tightening loopholes in current regulations are all needed to identify and eliminate the tobacco and vaping industry’s influence on policymaking. Given their previous track record, we emphasize the need to counter industry interference tactics with urgency to prevent a new generation of nicotine dependence and to support and protect future action in tobacco control.

**Contribution to Health Promotion**

- Tobacco industry interference tactics have been documented for decades and continue to be employed today by industry actors to delay or derail e-cigarette (vaping) policies.
- Commercial factors influence vaping uptake, reinforcing marketing activities, widespread supply of products and positive social norms and acceptability of vaping among young people.
- Investment in monitoring should be prioritized within all government tobacco and vaping control reform packages.
- Ongoing monitoring and research will help public health advocates and policymakers anticipate and counter future industry plans.

## INDUSTRY INFLUENCE IN THE YOUTH AND YOUNG ADULT VAPING CRISIS: IS HISTORY REPEATING ITSELF?

Public health efforts to reduce tobacco use are directly opposed to tobacco companies’ financial obligations to shareholders to increase profits. For decades, the tobacco industry has purposefully and strategically deployed tactics to derail, undermine and weaken public health policies in order to maintain and grow tobacco markets. Such interference, which has been documented widely, include: political lobbying to influence and block regulation; actively spreading disinformation and fabricating and discrediting scientific evidence to manipulate public opinion and intimidating governments with litigation (World Health Organization, 2023).

In recent decades, the industry has pivoted to claim moral and social accountability and trustworthiness. By promoting corporate social responsibility (CSR) initiatives and claims of moving away from combustible tobacco sales, the industry argues they are a legitimate stakeholder in reducing global tobacco deaths (Fooks *et al*., 2011). In truth, the tobacco industry is actively establishing and growing its new product pipeline to restore long-term market sustainability and shareholder confidence as combustible tobacco use declines (Evans-Reeves *et al*., 2020). Establishing a new generation of nicotine users through new nicotine products is critical for the industry, particularly given combustible smoking in younger age cohorts is lower than ever before (Edwards *et al*., 2022).

Vaping has grown rapidly among young people in recent years (Sreeramareddy *et al*., 2022). This has been perpetuated by industry-targeted youth-friendly product designs and advertising, and efforts to normalize their products while weakening policies to curb vape use (World Health Organization, 2021). The tactics employed today to delay or derail vaping policies, however, are not new. The industry continues to follow its old playbook, recycling interference strategies which proved effective in the past, whilst simultaneously developing new strategies, leveraging current communications opportunities and platforms.

Our article reviews the evidence on tobacco and vaping industry interference and demonstrates how these tactics have been used in the context of new nicotine products, with a primary focus on vaping products. An Australian case study is used to illustrate how this interference plays out in a real-time policy context. We argue that efforts to curb vaping among young people must occur alongside systematic monitoring of industry attempts to undermine such efforts, highlighting practical steps needed to eliminate industry influence on public health. The tobacco and vaping industry attempts to undermine public health policies and promote youth vaping also serves as a significant distraction to tobacco control efforts. It is therefore critical to also ensure continued action by governments in tobacco control policy and smoking cessation support, alongside any efforts to curb vaping among young people.

## INDUSTRY INTERFERENCE IN THE CONTEXT OF VAPING

### Shaping the evidence base

Historically, tobacco companies have manipulated scientific discourse by funding research to strategically serve their interests, as well as discrediting evidence about the health effects of tobacco use (World Health Organization, 2023). Unsurprisingly, this interference continues today in the context of e-cigarettes. Following the merger between Altria (the American parent company of Philip Morris USA) and JUUL labs (the US e-cigarette market leader), the research arm ‘JLI Science’ was established to ‘*expand the body of scientific knowledge around electronic nicotine delivery system products, which we believe can offer adult smokers a non-combustible alternative to combustible cigarettes and, in so doing, reduce the harm associated with tobacco use*’ (JUUL Labs Science, 2024). This research program has been scrutinized, with one independent review of JUUL Labs-sponsored clinical trials finding that only 46% of prespecified outcomes were reported or properly declared, raising concerns about selective outcome reporting bias (Nicholas *et al*., 2023). Another review of JUUL’s sponsored research found that it failed in seven of eight criteria for evaluating tobacco industry-supported research and that findings which favoured the company’s interests were promoted in the news media and at scientific meetings (Tan *et al*., 2019).

A systematic review of the literature on the potential harms of e-cigarettes investigated whether papers with an industry-related conflict of interest (COI) were associated with favourable results for the industry. It reported the odds of finding no harm were 66.92 times higher if the study was industry-related (Pisinger *et al*., 2019). Likewise, Vidaña-Perez *et al*. (2023) found that articles reporting a COI had a four times higher probability of having favourable results towards vaping.

Through the establishment of the Foundation for a Smoke-Free World (FSFW) in 2017 with a $1 billion commitment (USD), Philip Morris International (PMI) sought to shape a research program aligned with their commercial interests of furthering the use of their heated tobacco products and vapes (Evans-Reeves *et al*., 2020). Claiming to be ‘free from the control or influence of any third party’ (Tobacco Tactics University of Bath, 2023), a tax return and company document analysis showed that FSFW funds were largely used for public relations and communications aligning with their advocacy strategy (STOP—Stopping Tobacco Organizations and Products, 2023). The small proportion of research that was funded focused on ‘tobacco harm reduction’ through the use of industry products (Legg *et al.*, 2019, 2024; STOP, 2023). The FSFW and its grantees have also published research obscuring the involvement of PMI in the foundation, advocated for the renormalization of the tobacco industry and their involvement in science and policy and rallied against tobacco control advocates and researchers (Legg *et al.*, 2024).

### Corporate image and public relations

For over two decades, CSR has been key in the tobacco industry’s marketing strategies to improve its image (Smith and Malone, 2003). Such strategies include contributing to youth smoking prevention work (McDaniel *et al*., 2016), preventing and highlighting child labour practices (McDaniel *et al*., 2016), improving gender equality (McDaniel and Malone, 2009) and promoting environmental sustainability (Otanez and Glantz, 2011; Smith and McDaniel, 2011; McDaniel and Malone, 2012; McDaniel *et al*., 2016; Epperson *et al*., 2017). A review of the CSR activities described on tobacco company websites showed that their CSR promotions served as a lobbying strategy to build credibility, establish allies, influence policymakers, shape the tobacco control agenda and undermine tobacco control policy (McDaniel *et al*., 2016).

Building a positive corporate image is also critical for vaping corporations (which includes Big Tobacco) serving to position the vaping industry as a partner in efforts to reduce smoking under a harm reduction agenda. This serves to increase industry influence among public health policymakers and at times, even among the public health researchers and professionals through industry sponsorship and/or attendance at smoking cessation events and conferences. Vape starter kits are also now being provided to smokers under a UK government-led smoking cessation initiative (Wise, 2023). The vaping industry has invested in supposed youth prevention programs as a key component of CSR, reminiscent of tobacco companies’ disingenuous purported efforts to prevent youth smoking in past decades (Landman *et al*., 2002). Responding to public concern about increasing JUUL use among US youth, JUUL Labs announced a 3-year, $30 million (USD) underage vaping prevention program in 2018. A retrospective review of the campaign found that JUUL’s marketing expenditure promoting CSR exceeded their investment in youth prevention work, with the campaign not delivered in a way to reach parents. Corporate image expenditures, however, were focused on areas where JUUL’s promotional strategies were being investigated (Kostygina *et al*., 2022).

### Political engagement and lobbying

Leveraging their claimed revitalized corporate image, commercial vaping actors have argued that their expertise is required in policymaking decisions and that stakeholder engagement is a democratic right (Smith *et al*., 2010; Ikegwuonu *et al*., 2022). Challenging government policy and arguing for industry engagement in policy discussions is a strategy that PMI has proactively taken in more recent years. PMI’s leaked ‘10 year Corporate Affairs Objectives and Strategies’ document published by Reuters in 2014, stated a key objective as ‘establish PMI as a trusted and indispensable partner, leading its sector and bringing solutions to the table’, and to ‘define and pave the way for the right fiscal and regulatory frameworks to secure PMI’s RRP (reduced-risk products) portfolio as the pathway for future growth’ (Philip Morris International, 2014). In Australia, these strategies have played out as political marketing campaigns across media, active strategic political lobbying and establishing alliances with, and directly or indirectly funding, front groups that serve the industry’s interests.

### Youth-centred promotions and marketing

Vaping advertising and marketing are key in raising awareness of e-cigarettes and subsequently, influencing product experimentation and use. Research shows that exposure to e-cigarette marketing is associated with experimentation in adolescents and young adults (Collins *et al*., 2019). Between 2018 and 2019, JUUL spent US$57 million on television advertising (Struik *et al*., 2020). While the industry consistently argues that advertising is directed at adults who smoke, it is likely that these advertisements also attract youth (Struik *et al*., 2020). Perceptions of the relative safety or e-cigarettes have been perpetuated by e-cigarette marketing (McDonald and Ling, 2015), for example, describing e-cigarette aerosol as ‘water vapour’ and promoting e-cigarettes as ‘less harmful than cigarettes’. Adolescent and young adult exposure to vaping advertisements on social media may also lead to decreased risk perceptions about e-cigarettes as they normalize the use of e-cigarettes by promoting the social aspects of vaping (Grana and Ling, 2014; Pokhrel *et al*., 2019); and simply because they are particularly appealing to youth (Padon *et al*., 2017).

In Australia, there is evidence that PMI planned to target their heated tobacco product, IQOS, at young adults if their lobbying efforts were successful in allowing IQOS to be sold in Australia as a legal product. Former tobacco company employees in Australia revealed that PMI had attempted to launch their IQOS product as a ‘premium’ and ‘younger’ brand in Australia (Watts *et al*., 2020). Whilst simultaneously lobbying for the legalization of heated tobacco in Australia, PMI employees were establishing new tobacco retail agreements with carefully selected bars, clubs and pubs frequented by young people (Watts *et al*., 2020).

### New products and adaptations

The tobacco and vaping industry also strategically targets young people with appealing product features such as an array of flavours, design and packaging aesthetics, the ease of concealment of the products, product affordability and the presence of nicotine. Australian research has indicated that flavours are an important characteristic of e-cigarettes (Watts *et al*., 2022). Additionally, the enticing design of products, often resembling child-friendly packaging such as juice boxes or lollies (Fadus *et al*., 2019; Han and Son, 2020; Dormanesh and Allem, 2022) also appeal to young people. An increase in products designed for inconspicuous use (stealth vaping) (Dormanesh and Allem, 2022) can aid adolescents in hiding their vaping from teachers or parents.

Despite growing regulations to limit the appeal and supply of vapes to young people, the tobacco and vaping industry has adapted their products or attempted to introduce new products. Australian research showed that manufacturers were removing the word ‘nicotine’ from their products, despite nicotine being present in high concentrations, to circumvent regulations that only allowed non-nicotine vapes to be sold by retailers (Jenkins *et al*., 2024). In New Zealand, restrictions on disposable vapes including requiring a removable battery, a child safety mechanism and a cap on nicotine concentrations were implemented. However, in response, the vaping industry modified the design of its products to allow continued sales of its disposable products (Hardie *et al*., 2024). Shortly after the restrictions came into effect, retailers started promoting vapes with a removable battery for only $5 (NZD). This ‘whack-a-mole’ effect, where regulations to stamp out one product resulted in the industry releasing a modified product to circumvent the restrictions, is widespread. After the US Food and Drug Administration indicated they would prioritize enforcement of flavoured pod and cartridge e-cigarette device restrictions in 2020, disposable devices exploded in popularity. The industry also circumvented the restrictions on flavoured pod and cartridge devices by selling add-on flavour enhancers (Gaiha *et al*., 2022).

### An Australian case study

To demonstrate how these tactics play out in real-time, we outline examples of how tobacco and vaping industry actors in Australia have strategically deployed media and public relations campaigns, and used third-party allies in lobbying efforts, to weaken, delay and derail Australian vaping policy.

Box 1. **Vaping legislation in Australia**

From October 2021, Australian consumers of vaping products were required by law to hold a prescription from a health professional to legally purchase nicotine-containing vapes from pharmacies, or via the personal importation scheme (imported internationally) to support smoking cessation efforts (Therapeutic Goods Administration, 2021). However, non-nicotine vaping products were excluded from the legislation and were freely available at retail outlets for adults. This caveat enabled a loophole for manufacturers and industry, with vaping products incorrectly labelled as being ‘nicotine-free’ or nicotine content omitted completely to bypass the prescription pathway. The result was continued sales of nicotine vaping products by retailers, creating enforcement challenges as laboratory testing is required to distinguish nicotine from non-nicotine vapes—a costly and time-consuming activity.

In May 2023, the Australian government announced regulatory measures to restrict vaping access further, which were to be implemented across 2024 (Parliament of Australia, 2023). The first phase commenced in January 2024, banning the importation of all disposable, single-use vapes and the end of the personal importation scheme. Domestic controls on all vaping products followed the passage of legislation through the Senate in the Australian Federal Parliament in June 2024. From 1 October 2024, therapeutic vapes are available only in pharmacies as behind-the-counter medicines to patients aged 18 years and over (Therapeutic Goods Administration, 2024). It is now illegal for general retailers such as tobacconists and convenience stores to sell any type of vape, regardless of nicotine content. Vapes supplied behind the counter through pharmacies are also required to have minimum quality standards, such as nicotine concentrations, limited flavours and standardized medical-style plain packaging (Therapeutic Goods Administration, 2023).

## POLITICAL MARKETING AND THE STRATEGIC USE OF MEDIA

**Fig. 1:**
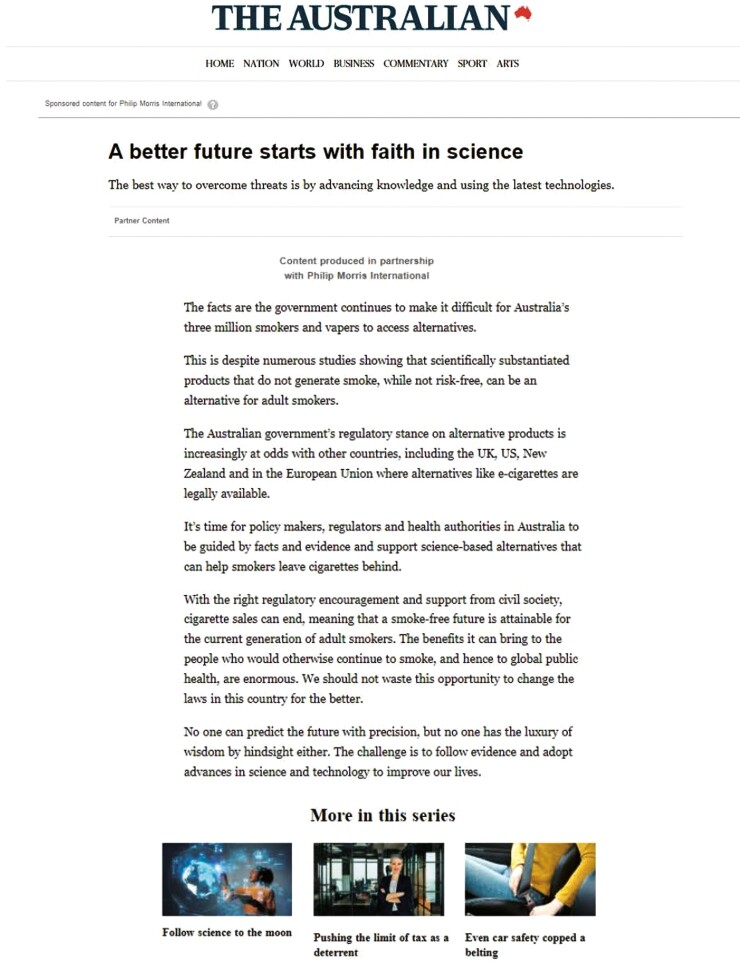
Sponsored advertorial by Philip Morris International in The Australian newspaper online (extract of full article) (Philip Morris International, 2020).

In November 2020, PMI paid for eight sponsored advertorials in The Australian newspaper framing the government’s strong stance on e-cigarettes as regressive and dismissive of science (Figure 1). They positioned the company as being on the cutting edge of technology needed to solve the world’s biggest problems (Philip Morris International, 2020). The industry’s deceptive narrative and pro-vaping messages have also dominated the social media landscape in Australia (McCausland *et al*., 2020). While Australia has strict tobacco advertising, promotion and sponsorship laws, tobacco and vaping industry campaigns with a political purpose are exempt from the Tobacco Advertising Prohibition Act (Australian Government, 2017).

**Fig. 2:**
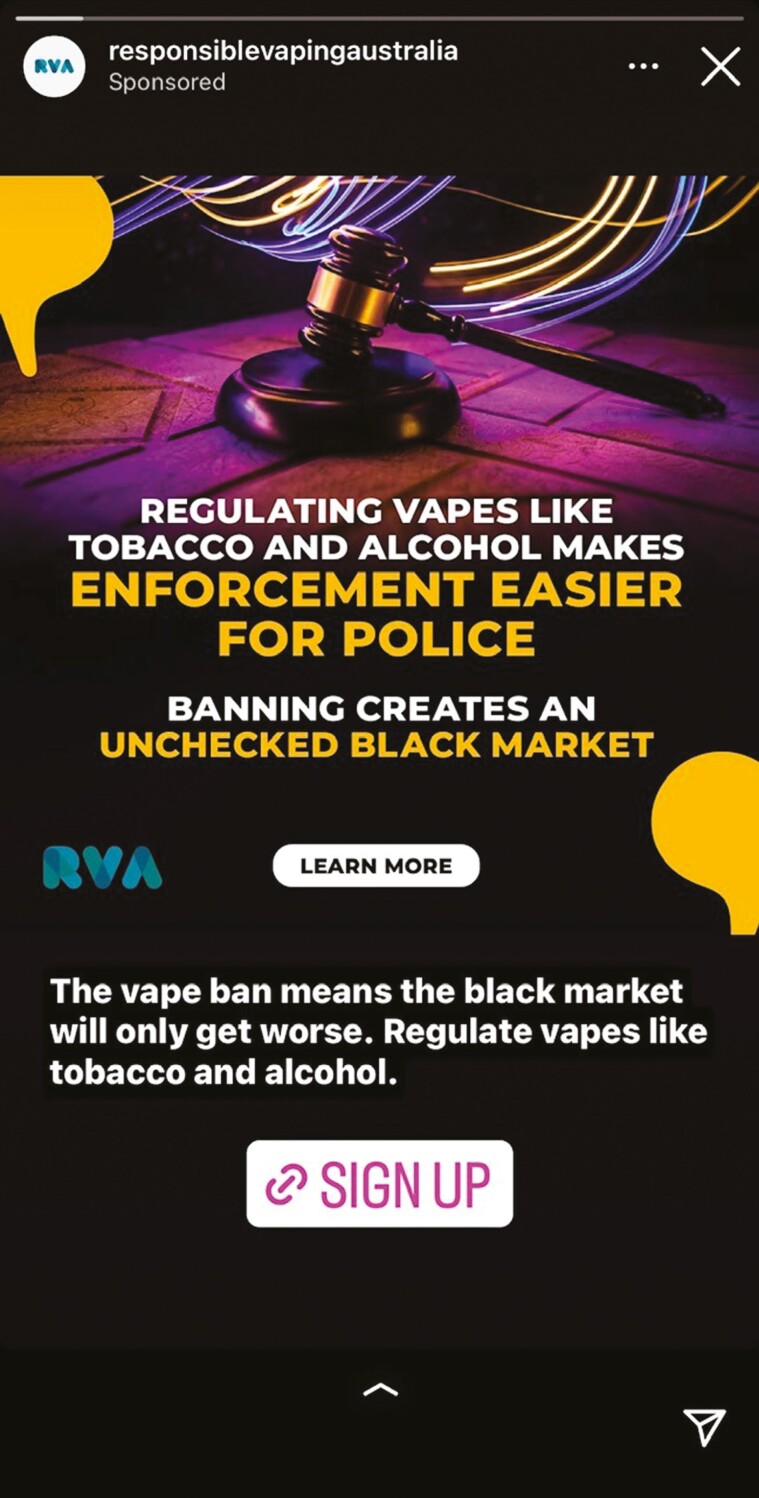
BAT’s Responsible Vaping Australia campaign sponsored advertisement on Instagram.

**Fig. 3:**
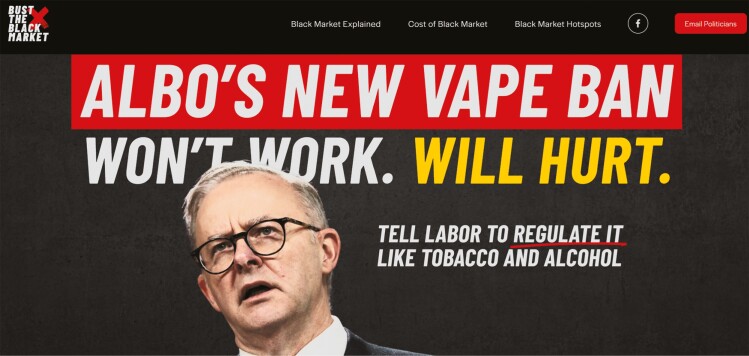
The Australian Taxpayers Alliance campaign ‘Bust the Black Market’ (Australian Taxpayers Alliance, 2024).

Like PMI, British American Tobacco (BAT) had skin in the game in efforts to block the 2024 Australian vaping reforms from passing the Australian Senate in June 2024. VEEV and Vuse are e-cigarettes made by PMI and BAT (respectively) and the proposed ban on all vaping products outside of pharmacy-only retail access would greatly restrict their Australian market prospects, with potential ripple effects globally. BAT launched ‘Responsible Vaping Australia’ immediately after the announcement of Australia’s 2024 vaping reforms and ran a series of advertisements opposing the laws. Whilst claiming to be an ‘education research centre’ representing Australian vape retailers, their advertisements on Meta heavily target young adults calling for the regulation of vapes as consumer goods, akin to alcohol and tobacco (Figure 2) (Davey, 2023b). The advertisements targeted Meta users with an interest in nightclubs, pubs, bars and clubbing subculture, and were run without any disclaimer of the link to BAT (Struik *et al*., 2020). Vaping lobby group ‘Australian Taxpayers Alliance’, which has links to both PMI and BAT, ran a similar campaign, ‘Bust the Black Market’ in 2024. It involved full-page advertisements in Australian newspapers calling for the government to regulate e-cigarettes like alcohol and tobacco (Figure 3) (May, 2024b). The ‘Australian Taxpayers Alliance’ also operates under different lobby groups, such as Legalise Vaping Australia (Legalise Vaping, 2024).

### Lobbying, alliances and front groups

Establishing a front group or enlisting third-party allies, like the ‘Australian Taxpayers Alliance’ and ‘Responsible Vaping Australia’ for political lobbying purposes is a long-established tobacco industry tactic to oppose public health reforms (Tobacco Tactics University of Bath, 2021). In Australia, this tactic is not easily tracked as it is poorly recorded on lobbyists’ registers (Watts *et al*., 2023). Tobacco companies strategically distance themselves from front groups, with funding often filtered through multiple avenues to obfuscate tobacco connections. One such example is PMI’s indirect use of the Australian Retailers Association (ARA) to lobby for the legalization of nicotine e-cigarettes as a commercial good. In 2020, the ARA signed a $250,000 (AUD) contract with global public relations firm Burson Cohn & Wolfe (BCW), with a possible 1-year $250,000 extension, specifically to lobby the government on e-cigarettes. BCW began working with the ARA in 2019, however, they also had contracts with PMI (Chenoweth, 2021a, 2021b; Safran, 2021). Other tobacco and vaping industry front groups in Australia include the Australasian Association of Convenience Stores (AACS) and Master Growers Association (MGA), both of which refused to disclose the value of tobacco and vaping industry funding they had received in a recent (2023) Australian senate inquiry (Davey, 2023a) (Tobacco Tactics University of Bath, 2022).

Tobacco companies are increasing political lobbying efforts in Australia aiming to legalize the over-the-counter retail sale of vaping and heated tobacco products. PMI, BAT and Imperial Tobacco have employed individuals engaged in lobbying the Australian government, with almost half of these employees having previously held government jobs, often within 1 year of working in public office (Watts *et al*., 2023). Known as the ‘revolving door’ this lobbying tactic allows the industry to gain and share insider knowledge about the policymaking process, develop close relationships with people of influence and establish quid pro quo contributions (Blanes i Vidal *et al*., 2012). Tobacco companies were found to be meeting with senior Australian government officials and politicians at key time points when pro-vaping lobbyists were trying to legalize nicotine e-cigarette consumer sales (Watts *et al*., 2023). PMI and BAT have also lobbied the federal government by making submissions to legislative reviews (Philip Morris Limited, 2019; British American Tobacco Australia, 2020; Watts *et al*., 2020), participating in inquiry hearings, holding private meetings with members of parliament, making political donations and sending letters to key people with political influence (Hutcheon and Bogle, 2019; Bogle, 2020; Chenoweth, 2021b).

## IDENTIFYING AND BLOCKING INDUSTRY INTERFERENCE—WHAT NEEDS TO BE DONE?

History shows that the tobacco industry responds to strong public health regulations with product adaptations and new strategies to secure and protect future profits and generate revenue streams. Globally, governments are grappling with how to control the rapidly growing use of vaping products by young people. Measures such as restricting retail supply, implementing educational campaigns, regulating products and greater enforcement of laws are all important components of a comprehensive approach to address the issue. However, it is also critical to recognize the influence of commercial factors on vaping uptake. These factors reinforce marketing activities, widespread supply of products and positive social norms and acceptability of vaping among young people. Given the tobacco and vaping industry’s concerted efforts to block and weaken public health policy, manipulate the public narrative and target their addictive products at vulnerable young people, identifying and eliminating commercial influences must be included within a comprehensive approach to reduce vaping uptake.

Continued monitoring of the industry’s activities and strategies, whilst holding the industry to account for such actions, is central to identifying and blocking tobacco and vaping industry interference. Ongoing monitoring and research will minimize the risk of being blindsided by the industry’s ‘next move’ and can help public health advocates and policymakers anticipate future industry plans. Industry interference is, in our opinion, the single biggest threat to ongoing innovation in tobacco control, and therefore investment in monitoring should be prioritized within all government tobacco and vaping control reform packages. While the focus of the tobacco and vaping industry appears to be on new nicotine products, it is critical for governments to also not lose sight of the importance of continued investment and action in tobacco control.

Achieving political transparency is equally important. Democratic systems can be undermined by influential corporations like tobacco companies. In Australia, the movement of tobacco lobbyists through the revolving door is rampant, despite being a party to the World Health Organization Framework Convention on Tobacco Control, which stipulates that policymaking must be protected from the vested interests of the tobacco industry (Article 5.3) (World Health Organization, 2008). At a bare minimum, details such as historical records of lobbyists, the companies they represent and details of their lobbying activities, including meeting attendees, dates and reasons for meetings must be made publicly available. Cooling-off periods between government and industry for people working in public office should also be independently policed and enforced.

Loopholes in current regulations need to be identified, closed and/or tightly regulated, aligned with the need for industry monitoring research. The tobacco and vaping industry is nimble and innovating in skirting product regulations, or other restrictions (such as marketing). With our knowledge of repeated industry tactics, we have an opportunity to act proactively rather than rely on reactive solutions which are easily circumvented. Comprehensive regulation of devices, such as a total ban on disposable products, is needed to ensure the industry is unable to skirt laws through clever marketing and product modifications. However, where comprehensive policies have been implemented, continued monitoring of industry activities, including what products continue to be sold and promoted is needed. In Australia, where the importation of disposable vapes has been banned as of January 2024, tobacco-free oral nicotine pouches have entered the market. Despite being illegal for sale by retailers, the products have been seized in recent government enforcement raids of Australian retailers (Theocharous, 2024), and promoted to young people via Tik Tok and Instagram influencer marketing (May, 2024a). In New Zealand, restrictions on product design, such as the requirement for removable batteries allowed the industry a foot in the door. Instead, a complete ban on disposable products better aligns with the public health agenda to reduce vaping uptake and prevalence. Likewise, comprehensive advertising, promotion and sponsorship bans should also include political marketing campaigns, particularly given the industry uses this loophole to purposefully undermine government policy.

The abundant evidence of previous tobacco industry interference gives us clear insight into how the industry behaves when faced with a threat to business. With governments increasingly implementing policies to deter young people from vaping, it is as important as ever to be on the front foot and call out industry interference tactics as they happen, and to act on them with urgency.
